# Supplementary material for: Assessment of Tilapia (Oreochromis spp.) Welfare in the Semi-Intensive and Intensive Culture Systems in Thailand
Source: Animals (Basel). 2023 Aug 2;13(15):2498. doi: 10.3390/ani13152498 (PMC10416865; doi:10.3390/ani13152498)

Supplementary Table S1. The assessment of health factors in tilapia (Pedrazzani et al., 2020).

Indicators	Score	Descriptions or Reference Values
Eyes	1	Apparently functional and healthy
	2	Haemorrhage, exophthalmos, traumatic injury; Unilateral
	3	Haemorrhage, exophthalmos, traumatic injury; Bilateral
	4	Bilateral cataract, chronic condition, impaired vision
Jaws	1	Normal aspect, healthy
	2	Light to moderate superior or inferior deformity
	3	Severe superior or inferior deformity (affecting feeding)
Operculum	1	Normal aspect, healthy
	2	Partially covering the gills (≥75% covered)
	3	Partially covering the gills (<75% covered)
	4	Unilateral or bilateral absence
Skin	1	Normal aspect, healthy
	2	Scar tissue, scale loss, ulcers, or superficial injuries <1 cm^2
	3	Ulcers or superficial injuries >1 cm^2, redness, light necrosis
	4	Severe necrosis, darkening, bleeding, inflammation
Fins	1	Normal, healthy appearance
	2	Scarred or slightly necrotic tissue
	3	Moderate injury or necrosis (thickening/splitting)
	4	Severe necrosis, bleeding, inflammation, exposure of the rays
Gills	1	Normal aspect, healthy
	2	Light injury or necrosis, thickening or splitting
	3	Moderate injury or necrosis, thickening or splitting
	4	Severe necrosis, bleeding, inflammation, pallor or darkening
Spine	1	Normal structure
	2	Lordosis or scoliosis, normal weight
	3	Lordosis or scoliosis, weight loss
Ectoparasite	1	No infestation
	2	Moderate infestation (≤5 parasites)
	3	Intense infestation (>5 parasites)
Mortality (%)	1	≤ 10%
	2	≤ 25%
	3	≤ 50%
	4	> 50%

Supplementary Table S2. The assessment of environmental factors in tilapia (Pedrazzani et al., 2020).

Indicators	Score	Descriptions or reference values
Temperature (°C)	1	25 – 32
	2	20 – 24
	3	33 – 37
	4	<20 or >37
pH	1	6.0 – 8.5
	2	5.5 – 5.9 or 8.6 – 8.9
	3	9.0 – 10.0
	4	<5.5 or >10.0
Transparency (cm)	1	25 – 40
	2	41 – 65
	3	<25 or >65
Oxygen saturation (mg/L)	1	5.29 – 7.18
	2	3.78 – 5.21
	3	2.27 – 3.7
	4	<2.27 or >7.18
Non-ionized ammonia (NH_3; mg/L)	1	0.00 – 0.05
	2	0.05 – 0.10
	3	> 0.10
Nitrite (NO_2^-; mg/L)	1	0.00 – 0.50
	2	0.51 – 1.00
	3	>1.00
Alkalinity (mg/L of $CaCo_3$)	1	30 – 100
	2	20 – 30 or 100 – 200
	3	<20 or >200
Shading (%)	1	Homogeneous shading; 20 – 30
	2	Homogeneous shading; 31 – 40
	3	Homogeneous shading: <20 or >40 or heterogeneous shading
Predators	1	Absence
	2	Controlled presence
	3	Uncontrolled presence
Interspecific inhabitants	1	Absence
	2	Controlled presence
	3	Uncontrolled presence
Stocking density*	1	Ideal to 10% overpopulation
	2	10 – 20% overpopulation
	3	> 20% overpopulation

The optimal stocking density for earthen ponds without aeration is 1–3 fish/m² for fish weighing 1–300 g., while aeration is 5 fish/m². For cages culture, the optimal stocking density are: 2,000–3,000 fish/m² for fish weighing 1–30 g, 1,000–1,500 fish/m² for fish weighing 30–300 g, and less than 1,000 fish/m² for fish weighing > 300–1,000 g.

Supplementary Table S3. The assessment of behavioural factors in tilapia (Pedrazzani et al., 2020)

**Management**	**Score**	**Criteria**
Feeding duration	1	Apprehension of all food in 180 to 360 second
	2	Apprehension of all food in 120 to 179 second
	3	Apprehension of all food in < 120 second
	4	No apprehension of all food or ≥ 360 second
Capture period	1	Normal swimming, no or low dorsal fins or body parts on surface
	2	Excited swimming behaviour, >20 dorsal fins or low body parts on surface
	3	Swimming in different directions or decreasing activity, fish stuck against net
	4	Many fish floating on side, explosion of body to air, exhaustion

Supplementary Table S4. The assessment of nutrition factors in tilapia between raising systems, adapted from previous study (Pedrazzani et al., 2020)

Raising system	Weight (g)	Age (days)	Stock density (fish/m²)		FCR	CP (%)
			No aeration or renew	Aeration or renew		
Earthen pond	1–30	40–80	20–30	40–50	0.8–1.0	36–40
	30–200	80–120	4–5	6–10	1.2–1.3	28–32
	200–1,000	>120	0.8–1.2	2–3	1.4–1.6	28–32
Cage	1–30	40–90	1,200–1,500		0.8–1.0	40
	30–200	90–120	450–500		1.2–1.4	32
	200–1,000	>120	100–150		1.6–2.0	32

Figure S1. Score distribution of eyes, jaw, operculum, skin, fins, gills and spine among eight farms. Score 1; green, score 2; yellow, score 3; orange and score 4; red

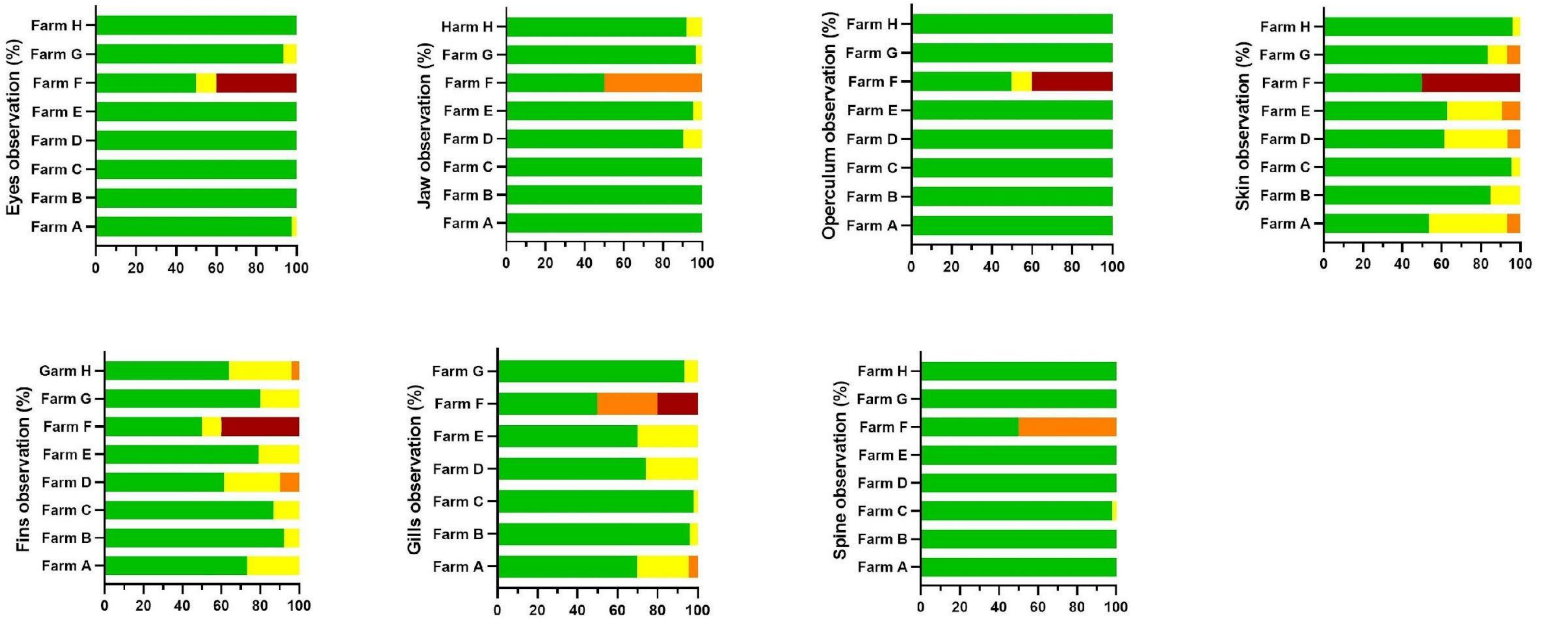

Supplement: Supplementary file 1 [file animals-13-02498-s001.zip › animals-2481871-supplementary.pdf]
